# Depression severity is associated with reduced pleasantness of observed social touch and fewer current intimate touch experiences

**DOI:** 10.1371/journal.pone.0289226

**Published:** 2023-08-02

**Authors:** Victoria C. Tinker, Paula D. Trotter, Charlotte Krahé

**Affiliations:** 1 Department of Primary Care and Mental Health, University of Liverpool, Liverpool, United Kingdom; 2 School of Psychology, Liverpool John Moores University, Liverpool, United Kingdom; Indiana University School of Medicine, UNITED STATES

## Abstract

Depression is associated with loss of pleasure in previously enjoyed activities and withdrawal from social interactions. Depression alters the perception of social cues, but it is currently unclear whether this extends to social touch. In the current cross-sectional study, we explored the association between depression severity, perceived pleasantness of observed social touch, and general longing for touch. For observed touch, we contrasted videos of slow touch (1-10cm/s), which optimally activates C tactile afferent nerve fibres and generally feels pleasant, with ‘non-CT-optimal’ touch (i.e., outside the 1-10cm/s range, commonly rated more neutral). We predicted that greater depression severity would be related to lower pleasantness ratings specifically for CT-optimal touch, and less longing for touch. *N* = 226 adults completed self-report measures of depression severity and longing for touch, and rated touch pleasantness for six videos depicting social touch at three velocities (3cm/s in the CT-optimal range, 0.5 and 30cm/s outside this range) and at two locations varying in CT innervation (palm vs. arm). We controlled for general anhedonia and individual differences in touch experiences and attitudes. Across touch locations, greater depression severity was associated with lower perceived pleasantness of touch, especially for the fastest non-CT-optimal (rather than the CT-optimal) velocity, contrary to our prediction. However, when grouping participants into probable vs. no/minimal depression, the probable depression group rated *both* the fastest non-CT-optimal and the CT-optimal velocity as less pleasant than did the no/minimal depression group. Overall, while depression was associated with perceived pleasantness of observed touch, this was not specific to CT-optimal touch. Furthermore, touch longing was not associated with depression severity. Instead, variance in depression symptoms was better explained by reduced levels of current intimate touch. Though the direction of causality is unclear, greater depression severity is related to lower pleasantness of observed social touch, and lower levels of current intimate touch.

## Introduction

Sustained low mood and a loss of interest and pleasure in previously enjoyed activities (anhedonia) are hallmark features of depression [1, 2]. These core symptoms, along with somatic symptoms (changes in sleep, appetite), and cognitive symptoms (e.g., concentration problems and feelings of low self-worth) are associated with distress and difficulties functioning in everyday life. In particular, depression can have a marked impact on social functioning [3], including withdrawing from social activities. Individuals with depression do not find social interactions as rewarding as their non-depressed counterparts [4, 5] and interpret social information in a negative way, leading to feelings of rejection and avoidance of social interactions [6]. In addition, altered perception of social cues has been reported in depression, such as reduced ability to recognise emotions from facial expressions [3]. However, it is unclear whether differences in the perception of social cues extend to social touch, a key feature of interpersonal relationships. Accordingly, in the present paper, we examined associations between depression symptoms and the perception of social touch, also considering levels of general anhedonia.

In particular, we focused on a specific type of social touch, namely slow, gentle, caress-like motions, termed ‘affective touch’ because it usually feels pleasant, and which plays an important role in interpersonal communication [7], social bonding [8], and physical and emotional wellbeing [9, 10]. Feelings of pleasantness associated with affective touch are mediated in part by the activation of unmyelinated C-tactile afferents (CT fibres or CTs). These fibres are present in hairy skin, for example the forearm [11–14], with little evidence of these afferents in the non-hairy (glabrous) skin of the palms of the hands [15] and soles of the feet [16]. Moreover, CTs are sensitive to the speed of touch, being optimally activated at stroking speeds of 1–10 cm/s, at which speeds touch is also perceived as most pleasant [17]. The response of CTs has been shown to be reduced by repeated stimulation, as well as rapid (> 10 cm/s) and very slow (< 1cm/s) stroking [17], with these velocities being generally perceived as more neutral in valence (neither pleasant nor unpleasant) than CT-optimal velocities. Specifically, an inverted U shape characterises the relationship between touch pleasantness and stroking velocity [17], demonstrating that touch is especially pleasant at slow speeds between 1–10 cm/s, and not at speeds below or above this range. Parents spontaneously stroke their infants–and adult romantic partners each other–at speeds consistent with optimal CT-activation [18–20], suggesting a key function in social bonding and connectedness [21, 22]. In addition, CT-optimal touch carries an appetitive value, encouraging approach motivation when paired with social stimuli [23].

Despite these general prosocial functions of affective touch, the perceived pleasantness of slow, gentle touch is shaped by context and individual differences (see [24]). For example, lower touch exposure (not receiving touch very often) is related to reduced pleasantness of CT-optimal touch [25] and reduced differences in pleasantness ratings between CT-optimal and non-CT-optimal velocities (a flattening of the U-shaped curve outlined above). Furthermore, individual differences in attachment style [26], notably a more insecure (anxious) attachment style characterised by perceived unreliability of others to help in times of need, is also linked to reduced pleasantness discrimination between CT-optimal vs. non-CT-optimal touch. Adverse early childhood experiences, such as neglect, are linked to the development of depression (see [27]), and individuals with depression have often experienced low touch exposure [28]. Furthermore, adults who have spent time in care, and who have experienced significantly greater childhood trauma and neglect than non-care-experienced people, show altered responses to CT-optimal touch, with a flattening of the inverted U shaped curve [29].

In regards to depression specifically, Triscoli et al. [30] found that individuals scoring more highly on a depression measure showed a less positive attitude towards social touch, based on a social touch questionnaire. Furthermore, Crucianelli and colleagues [31] recently provided evidence for a flattened U-shaped curve in depression. However, their study did not control for general loss of pleasure or touch experiences and attitudes (such as current intimate touch; childhood touch; attitude to self-care; attitude to intimate touch; and attitude to unfamiliar touch), making it difficult to ascertain whether depression severity is associated with reduced discrimination between affective and neutral touch over and above such factors. In the current study, we thus controlled for general anhedonia and touch experiences and attitudes.

Furthermore, although more severe depressive symptoms are linked to less positive attitudes towards social touch [30] and potentially reduced pleasantness of CT-optimal touch [31], it is unclear whether this extends to how much touch is desired. In general, lack of intimacy, and lack of other forms of touch, may result in a longing for touch [32]. Beßler et al. [32] studied “touch frequency” and “touch wish” in relation to different forms of interaction (such as hug, stroke, handshake etc.) and different interaction partners. For a quarter of their sample, touch frequency was lower than touch wish, indicating touch longing. Notably, Beßler et al. [32] focused on touch in general. When considering CT-optimal touch, research suggests that the more pleasant such ‘affective touch’ is perceived to be, the more an individual wants the touch [33, 34]. Individuals with less exposure to touch rate affective touch as less pleasant, but it is unclear whether this means it is less desired [25]. While lower perceived pleasantness ratings may be linked to reduced longing for touch, this has not yet been explored in relation to varying levels of depression.

Thus, the present study aimed to explore the association between depression severity and the perception of observed CT-optimal (vs. non-CT-optimal) touch in different locations, and touch longing. Individuals do not have to directly receive touch to experience feelings of pleasantness. Morrison et al. [35] showed participants videos of affective touch on the forearm (3 cm/s skin stroking) and found a similar response in the posterior insula to experiencing touch first-hand, identifying that the brain is similarly activated by vicarious and felt touch. Furthermore, Walker et al. [36] showed participants videos depicting social touch at CT-optimal and non-CT-optimal velocities and asked participants to rate the pleasantness. For this observed touch, the same pattern of pleasantness ratings was found (inverted U-shaped relationship with velocity) as previously seen with directly experienced touch [17, 29]. However, effects of location (i.e., greater preference for CT-optimal touch when applied to hairy CT-innervated compared to glabrous skin) in such vicarious paradigms are mixed and warrant further investigation. We thus included both the arm (hairy) and palm of the hand (glabrous) as touch locations in our study.

In sum, in our study, conducted during the Covid-19 pandemic, we recruited participants with varying levels of depression from the general population. Participants saw videos of stroking touch at velocities within and outside the CT-optimal range at two different locations (varying in CT innervation; palm vs. forearm) and rated the pleasantness of the touch. They also completed a measure of touch longing as a second dependent variable. Additionally, we captured touch experiences and attitudes, as well as general loss of pleasure, as potential confounding variables. Considering previous findings showing individuals with depression have experienced low touch exposure [28], and individuals with low touch exposure rate CT-optimal touch as less pleasant [25], we hypothesised that higher levels of depression severity would be associated with reduced pleasantness ratings for observed social touch, and especially for stroking in the CT-optimal range (1-10cm/s) administered to the (CT-innervated) arm (Hypothesis 1). Furthermore, we hypothesised that lower longing for touch would be associated with higher levels of depression severity (Hypothesis 2).

## Methods

### Design

The design and plan of analysis were pre-registered on the Open Science Framework (registered July 2021; https://doi.org/10.17605/OSF.IO/QTZ5R). A within-subjects design was used for the touch videos. Participants saw all conditions (six touch videos), but the order of velocities and locations were randomised across participants. Location had two levels: forearm (hairy skin location) and palm of hand (glabrous skin location), and velocity had three levels (as in [36]): one CT optimal (3cm/s) and two CT-non optimal (0.5 cm/s and 30 cm/s). Depression severity, measured by the BDI-II, was included as a continuous predictor variable and the interaction with velocity and location on pleasantness ratings examined. Longing for touch, our other dependent variable, was measured using a self-report questionnaire. We also measured general loss of pleasure (anhedonia), and experiences and attitudes to touch, to control for any impact these variables may have on the perception of observed affective touch.

### Participants

The sample consisted of 262 participants who provided complete data. Participants were members of the general public who were fluent in English, did not experience allodynia (find innocuous stimuli to be painful), and did not have any visual impairments. The sample consisted of 54 male and 208 female participants, with an average age of 36 years (*SD =* 10.7, range 19–80 years). The self-reported ethnic background was Caucasian (93%), African Caribbean (2%), Asian (4%) and Multi-ethnic (1%). Sixty (22.9%) participants reported to have received a diagnosis of depression at some point in their lives. Of these, 11 (18.3%) also reported to have received an additional and/or primary psychiatric diagnoses other than depression (including varying anxiety disorders, eating disorders, schizophrenia and personality disorder). Furthermore, *n* = 42 lived alone, *n* = 136 lived with one other person, *n* = 48 lived with two other people, *n* = 29 lived with three other people, and *n* = 7 lived with four other people. We collected this information as the study was conducted during the Covid-19 pandemic, where people were asked to stay at home and avoid socialising outside their household.

### Measures and materials

#### Pleasantness ratings of touch videos

Six videos (as used in [36]) depicting stroking touch at 2 locations (the forearm and palm of the hand), each at 3 different velocities (0.5, 3 and 30 cm/s) were shown. After each video, the question, “*How pleasant would it be to be touched like this*?” was presented, with participants indicating their response on a visual analogue scale with anchors “0 = ‘not at all pleasant’” to “100 = ‘extremely pleasant’”. Videos are available at: https://www.youtube.com/channel/UCgVzB3t6NCKwCFAX9Mr-_Lg/videos.

#### Longing for touch measure: Interpersonal Touch Picture Questionnaire

The LITPQ [32] is a measure of touch frequency and touch longing in which six different types of touch are visually presented in relation to different interaction partners, e.g., “to a romantic partner” or “to a male stranger”. Presented types of touch include hugging, stroking, kissing, holding, random touch, and shaking hands. In relation to each picture and interaction partner, participants are asked, “How often did you experience this type of touch in the last week?” (touch frequency) and, “How often would you have wanted to experience this type of touch in the last week?” (touch wish). Participants are asked to choose a value between zero and infinite. A longing for touch score was calculated by dividing (across interaction partners) touch wish by touch frequency, the resulting outcome therefore reflects a ratio of the two subscales. Values higher than one (LITPQ score *>* 1) are interpreted as longing for touch, because the desired amount of touch was not met. Values lower than one (LITPQ scores *<* 1), on the other hand, are seen as touch satisfied.

#### Beck Depression Inventory (BDI-II)

The BDI-II [37] is a 21-item self-report questionnaire for evaluating the severity of depression in general and psychiatric populations. Affective, cognitive, somatic, and vegetative symptoms are covered on a 4-point scale ranging from 0 (symptom absent) to 3 (severe symptoms). Items are summed to produce a total score (0–63). Higher scores denote greater depression severity. In the present study, Cronbach’s alpha for the BDI-II was α = .96.

#### Snaith–Hamilton Pleasure Scale (SHAPS)

The SHAPS [38] is a 14-item scale measuring anhedonia, or the inability to experience pleasure. The questionnaire includes statements about general activities, such as, “I would enjoy my favourite television or radio programme”, requiring a selection of “strongly disagree”, “disagree”, “agree”, or “strongly agree”. Either of the ‘disagree’ responses are scored as 1 point, and either of the ‘agree’ responses are scored as 0 points. A score of 2 or less constitutes a “normal” score, while an “abnormal” score is defined as 3 points or more. The SHAPS has adequate validity, satisfactory test-retest reliability [39], high internal consistency [39], and is a reliable, valid, and unidimensional instrument in adult outpatients with depression [40]. In the present study, Cronbach’s alpha for the SHAPS was α = .81.

#### Touch Experiences and Attitudes Questionnaire (TEAQ)

The TEAQ [41] is a 57-item self-report questionnaire measuring attitudes towards, and experiences of, positive social touch. The questionnaire consists of the following subscales: friends and family touch (FFT); current intimate touch (CIT); childhood touch (ChT); attitude to self-care (ASC); attitude to intimate touch (AIT); attitude to unfamiliar touch (AUT). Statements about touch experience or attitudes, such as, “I usually hug my family and friends when I am saying goodbye” require responses on a scale ranging from “disagree strongly” (1) to “agree strongly” (5). Eight of the 57 items are negatively worded and reverse scored. Higher scores on the TEAQ subscales denote more positive attitudes or more experiences of touch. Trotter et al. [41] demonstrated the TEAQ to have good face validity, internal consistency, construct validity in terms of discriminant validity, known-group validity and convergent validity, and criterion-related validity in terms of predictive validity and concurrent validity. In the present study, Cronbach’s alphas were α = .93 for FFT, α = .92 for CIT, α = .91 for ChT, α = .78 for ASC, α = .92 for AIT, and α = .78 for AUT, indicating good to excellent internal consistency for the TEAQ subscales.

### Procedure

Ethical approval was granted by the University of Liverpool Research Ethics Committee. Participants were invited to complete a study exploring the association between mood and experiences, perception, and attitudes related to social touch e.g., holding hands, hugging and arm stroking. The study was hosted on the online survey platform Qualtrics and was advertised via social media, such as Facebook, Gumtree, and online adverts through the University of Liverpool and Liverpool John Moores University. Data was collected from September to October 2021, that is, during the Covid-19 pandemic, during which varying degrees of social restrictions were imposed. Participants accessed the study via a link from the online advert. Participation consisted of the completion of demographic questions (age, sex, ethnicity, marital status, number of co-habitants, diagnosis of depression and/or other psychiatric disorders), followed by the BDI-II. Individuals then viewed the 6 videos (presented in a random order) and rated perceived pleasantness of the observed touch in each video. Lastly, participants completed the remaining measures, the order of which was randomised across participants: SHAPS [38], TEAQ [41], and LITPQ [32]. At the end of the survey, participants were presented with a unique code to claim a £5 Love2Shop voucher for their participation. The authors did not have access to information that could identify individual participants during or after data collection. The full study took approximately 20 minutes to complete.

### Data analysis plan

The plan of analysis was pre-registered on the Open Science Framework (https://doi.org/10.17605/OSF.IO/QTZ5R). Descriptive statistics included computing summary data for depression severity, perceived pleasantness ratings, and longing for touch ratings. As pleasantness ratings for the different speeds and locations were nested within individuals (a fully within-subjects design was employed), linear mixed modelling was implemented to test Hypothesis 1. Participant ID was included as a random effect. Fixed predictors were velocity (3 levels), location (2 levels), and depression severity, as well as all interaction terms. General loss of pleasure and experiences and attitudes to touch were included as covariates. In an additional exploratory analysis (not pre-registered), we re-ran the above analysis but grouped participants into probable depression (reporting a depression diagnosis or a BDI-II score ≥ 20) vs. no/mild depression (no depression diagnosis or a BDI-II score < 20; see *Descriptive statistics* below). To test Hypothesis 2, a multiple hierarchical regression analysis was carried out to explore the association between longing for touch and depression severity. Longing for touch was added in step 1, and general loss of pleasure and experiences and attitudes to touch were added in step 2. Depression severity was the outcome variable in this analysis.

## Results

Statistical analyses were conducted in IBM SPSS Statistics for Macintosh, Version 27.0 and Stata 16 [42]. The data file can be found here: https://osf.io/rbkxf/ (DOI 10.17605/OSF.IO/RBKXF).

### Descriptive statistics

Descriptive statistics are presented in Table 1. Depression severity ranged from 0–52, with a mean of *M* = 11.37 (*SD =* 11.28), falling in the ‘minimal mood disturbance’ range. BDI-II score was not correlated with age (*r*_*s*_ = 0.05, *p* = .386), and there were no sex difference in depression severity (Mann Whitney U test = 1.89, *p* = .059; male participants: *M* = 9.76, *SD =* 11.00; female participants: *M* = 11.79, *SD =* 11.34). In non-clinical populations, scores above 20 indicate moderate depression. In our sample, *n* = 56 (21.4%) participants had a BDI-II score of 20 or over. As 60 participants reported having had received a diagnosis of depression at some point in their lives, we also computed how many people had a diagnosis of depression *or* scored 20 or over on the BDI-II; 83 (31.7%) participants fell into this ‘probable depression’ group.

**Table 1:** Descriptive statistics for the full sample (*N* = 262).

	Measure	Mean (*SD*)	Median	Minimum	Maximum
	Depressive symptoms (BDI-II)	11.4 (11.3)	8.0	0.0	52.0
	General loss of pleasure (SHAPS)	1.1 (2.0)	0.0	0.0	11.0
	Longing for touch (LITPQ)	0.4 (0.6)	0.3	0.0	8.1
TEAQ subscales	Friends and family touch (FFT)	3.7 (1.0)	3.9	1.0	5.0
	Current intimate touch (CIT)	3.4 (1.0)	3.6	1.0	5.0
	Childhood touch (ChT)	3.9 (0.9)	4.0	1.1	5.0
	Attitude to self-care (ASC)	3.8 (0.9)	4.0	1.0	5.0
	Attitude to intimate touch (AIT)	4.1 (0.8)	4.2	1.2	5.0
	Attitude to unfamiliar touch (AUT)	2.7 (0.9)	2.6	1.0	5.0
Pleasantness ratings	Palm at 30cm/s	47.0 (30.7)	46.5	0.0	100.0
	Palm at 3cm/s	60.1 (27.8)	65.5	0.0	100.0
	Palm at 0.5cm/s	53.6 (30.3)	59.0	0.0	100.0
	Forearm at 30cm/s	46.2 (29.2)	43.5	0.0	100.0
	Forearm at 3cm/s	63.0 (28.0)	70.0	0.0	100.0
	Forearm at 0.5cm/s	54.8 (39.8)	60.0	0.0	100.0

Note. BDI-II = Beck Depression Inventory II; SHAPS = Snaith–Hamilton Pleasure Scale; LITPQ = Interpersonal Touch Picture Questionnaire; TEAQ = Touch Experiences and Attitudes Questionnaire.

### Correlations between self-report measures

Correlations between the self-report measures are presented in Table 2. Non-parametric Spearman’s Rho correlations were used, as depression scores were not normally distributed.

**Table 2:** Spearman correlations between self-report measures.

	BDI-II	SHAPS	TEAQ FFT	TEAQ CIT	TEAQ ChT	TEAQ ASC	TEAQ AIT	TEAQ AUT	LITPQ
BDI-II	-								
SHAPS	.46**	-							
TEAQ FFT	-.35**	**	-						
TEAQ CIT	-.42**	-.40**	.64**	-					
TEAQ ChT	-.33**	-.31**	.62**	.56**	-				
TEAQ ASC	-.20**	-.25**	.46**	.34**	.21**	-			
TEAQ AIT	-.24**	-.23**	.56**	.64**	.50**	.34**	-		
TEAQ AUT	-.08	-.04	.31**	.16**	.36**	-.09	.30**	-	
LITPQ	-.12*	-.01	.20**	.22**	.13*	.11	.38**	.23**	-

Note

* *p* < .05

** *p* <. 01. BDI-II = Beck Depression Inventory II; SHAPS = Snaith–Hamilton Pleasure Scale; TEAQ = Touch Experiences and Attitudes Questionnaire: FFT = Friends and Family Touch; CIT = Current Intimate Touch; ChT = Childhood Touch; ASC = Attitude to Self-Care; AIT = Attitude to Intimate Touch; AUT = Attitude to Unfamiliar Touch; LITPQ = Interpersonal Touch Picture Questionnaire.

There was a moderate and significant positive correlation between the BDI-II and the SHAPS (see Table 2), indicating that higher levels of depression symptoms were related to a greater general loss of pleasure. There was also a weak, but significant negative correlation between the BDI-II and LITPQ, indicating that higher levels of depression symptoms were related to less longing for touch. This was explored further in the regression analysis (see below), in which we controlled for touch experiences and attitudes and general loss of pleasure.

The BDI-II and the majority of TEAQ subscales were significantly negatively correlated (see Table 2), indicating that greater depression severity was related to less positive attitudes and/or experiences of friends and family touch, current intimate touch, childhood touch, attitude to self-care, and attitudes to intimate touch. The subscale capturing attitudes to unfamiliar touch, however, was not significantly correlated with the BDI-II.

### The association between depression severity and the perception of observed social touch

A linear mixed model was run to examine the interaction of depression severity, touch velocity, and touch location on perceived pleasantness of observed touch, controlling for TEAQ and SHAPS scores. Full model results are presented in Table 3.

**Table 3:** Linear mixed modelling results predicting perceived pleasantness of observed touch.

			Levels	*b*	*SE*	*p*	95% CIs		Wald *χ*^*2*^ test
Depression severity				-0.52	0.16	.001	-0.83	-0.21	
Velocity		0.5cm/s		-8.26	2.20	< .001	-12.58	-3.94	*χ*^*2*^(2) = 31.94, *p* < .001
		30cm/s		-12.20	2.20	< .001	-16.52	-7.88	
Location		Palm		-1.23	2.20	.576	-5.55	3.09	
Location x depression severity		Palm		-0.15	0.14	.275	-0.42	0.12	
Velocity x location		Palm at 0.5cm/s		0.06	3.12	.984	-6.05	6.17	*χ*^*2*^(2) = 0.32 *p* = .852
		Palm at 30cm/s		1.56	3.12	.618	-4.55	7.66	
Velocity x depression severity		0.5cm/s		0.00	0.14	.978	-0.27	0.27	*χ*^*2*^(2) = 11.51, *p* = .003
		30cm/s		-0.40	0.14	.003	-0.67	-0.13	
Location x velocity x depression severity		Palm at 0.5cm/s		0.15	0.19	.437	-0.23	0.53	*χ*^2^(2) = 1.04, *p* = .595
		Palm at 30cm/s		0.19	0.19	.338	-0.20	0.57	
	TEAQ		FFT	0.75	.17	< .001	0.41	1.09	
			CIT	-0.11	.14	.437	-0.37	0.16	
			ChT	-0.56	.19	.003	-0.93	-0.19	
			ASC	0.88	.32	.007	0.24	1.51	
			AIT	0.63	.16	< .001	0.31	0.95	
			AUT	-0.14	.29	.634	-0.71	0.43	
	SHAPS			0.54	.73	.457	-0.89	1.98	
	Intercept			14.84	8.84	.093	-2.48	32.16	

Note. SHAPS = Snaith–Hamilton Pleasure Scale; TEAQ = Touch Experiences and Attitudes Questionnaire: FFT = Friends and Family Touch; CIT = Current Intimate Touch; ChT = Childhood Touch; ASC = Attitude to Self-Care; AIT = Attitude to Intimate Touch; AUT = Attitude to Unfamiliar Touch.

Depression severity was significantly associated with pleasantness ratings, indicating that greater depression severity was related to lower overall pleasantness ratings. There was also a significant effect of velocity on pleasantness ratings: consistent with previous findings [17], touch in the CT-optimal range (3cm/s: *M* = 61.54, *SE =* 1.34) was rated as more pleasant than both non-CT-optimal touch velocities (0.5cm/s: *M* = 54.21, *SE =* 1.34; 30cm/s: *M* = 46.60, *SE =* 1.34; Bonferroni-corrected pairwise comparisons: 0.5cm/s vs. 3cm/s *p* < .001, 30cm/s vs. 3cm/s *p* < .001, 30cm/s vs. 0.5cm/s *p* = .121). There was no significant effect of location.

**Fig 1:**
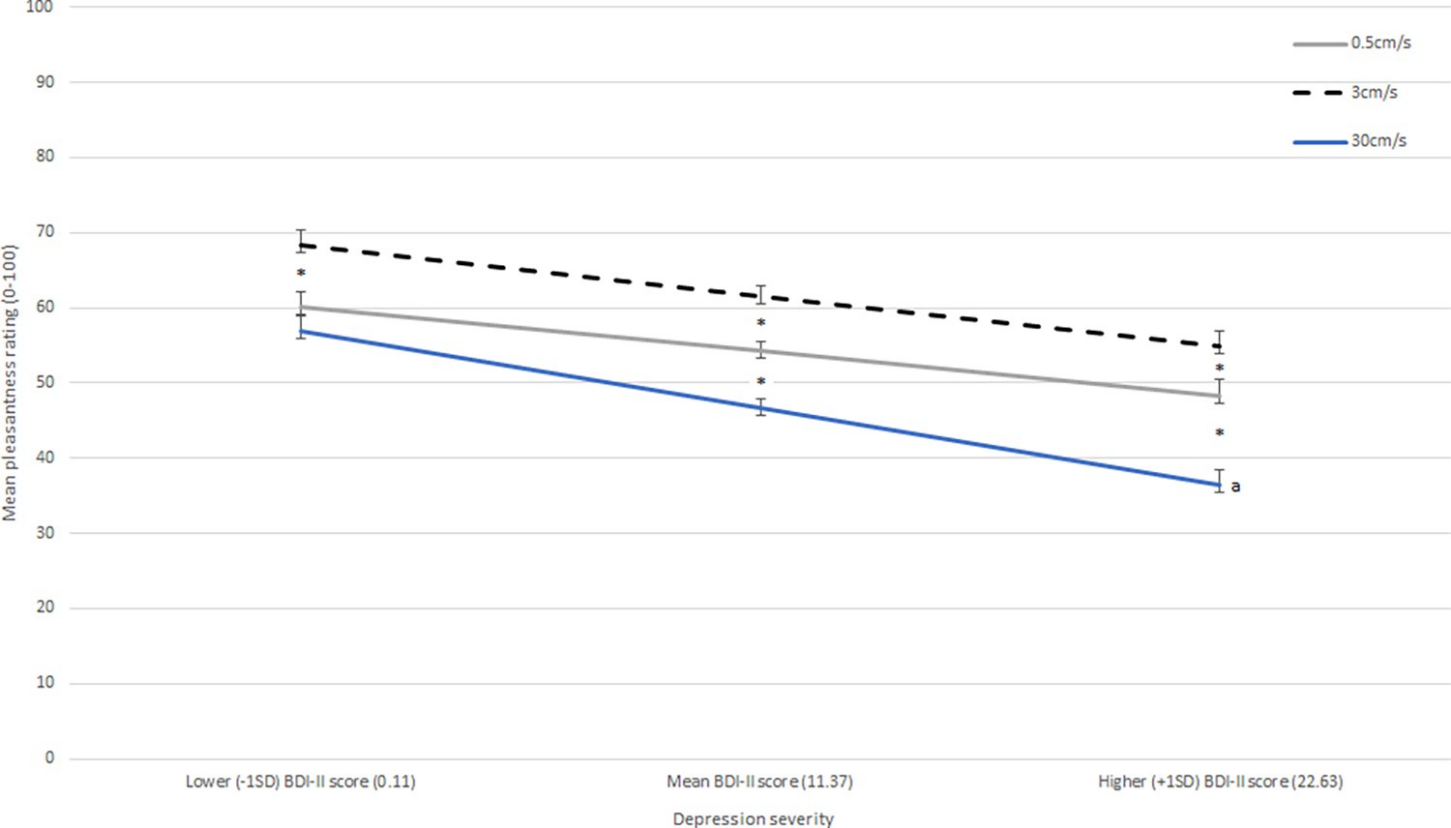
Interaction of stroking velocity and depression severity on pleasantness ratings (controlling for general anhedonia and touch experiences and attitudes). Error bars show ±1 standard error of the mean. BDI-II = Beck Depression Inventory II. * = significant difference between pleasantness ratings at this level of depression severity; a = significant difference in steepness of the 30cm/s slope versus the 0.5cm/s and 3cm/s slopes.

Examining interactions, only the velocity by depression severity interaction was significant (see Fig 1). None of the other 2-way interactions nor the 3-way interaction were significant–that is, there were no interaction effects with location.

Following up the velocity by depression interaction, Bonferroni-corrected contrasts (velocity at -1*SD*, mean, and +1*SD* of depression symptoms) showed that at *lower (-1SD) depression severity*, the CT-optimal velocity 3cm/s affective touch velocity was rated as significantly more pleasant than the non-CT-optimal velocities (0.5 cm/s contrast = 8.22; *SE =* 1.55, *p* < .001; 30cm/s contrast = 11.46, *SE =* 1.55, *p* < .001) and there was no significant difference between the two non-CT-optimal velocities (0.5cm/s vs. 30cm/s contrast = 3.24; *SE =* 1.55, *p* = .221). At *mean levels of depression symptoms*, the CT-optimal velocity 3cm/s affective touch velocity was also rated as significantly more pleasant than the non-CT-optimal velocities (0.5 cm/s contrast = 7.32; *SE =* 1.10, *p* < .001; 30cm/s contrast = 14.94, *SE =* 1.10, *p* < .001) and 0.5cm/s was also rated as significantly more pleasant than 30cm/s (contrast = 7.62; *SE =* 1.10, *p* < .001). Lastly, at *higher (+1SD) depression severity*, the CT-optimal velocity 3cm/s affective touch velocity was again rated as significantly more pleasant than the non-CT-optimal velocities (0.5 cm/s contrast = 6.43; *SE =* 1.55, *p* < .001; 30cm/s contrast = 18.43, *SE =* 1.55, *p* < .001), and 0.5cm/s was also rated as significantly more pleasant than 30cm/s (contrast = 12.00; *SE =* 1.55, *p* < .001). Thus, our first hypothesis was only partially supported. While we found that higher levels of depression symptoms were associated with lower perceived pleasantness across all three velocities, this was especially evident for the fastest non-CT-optimal velocity rather than the CT-optimal velocity. Indeed, in examining the steepness of the slope for each velocity, the 30cm/s slope was significantly steeper across levels of depression severity than the 3cm/s (contrast = -.31, Bonferroni-corrected *p* = .004) and 0.5cm/s slopes (contrasts = -.39, Bonferroni-corrected *p* < .001). The slopes for 3cm/s and 0.5cm/s did not differ from each other (contrast = .08, *p* = .999). Thus, the interaction between depression symptoms and stroking velocity was driven by the difference between the two non-CT-optimal velocities and specifically the fast non-CT-optimal velocity being rated much as much less pleasant than the very slow non-CT-optimal velocity at higher levels of depression severity.

**Fig 2:**
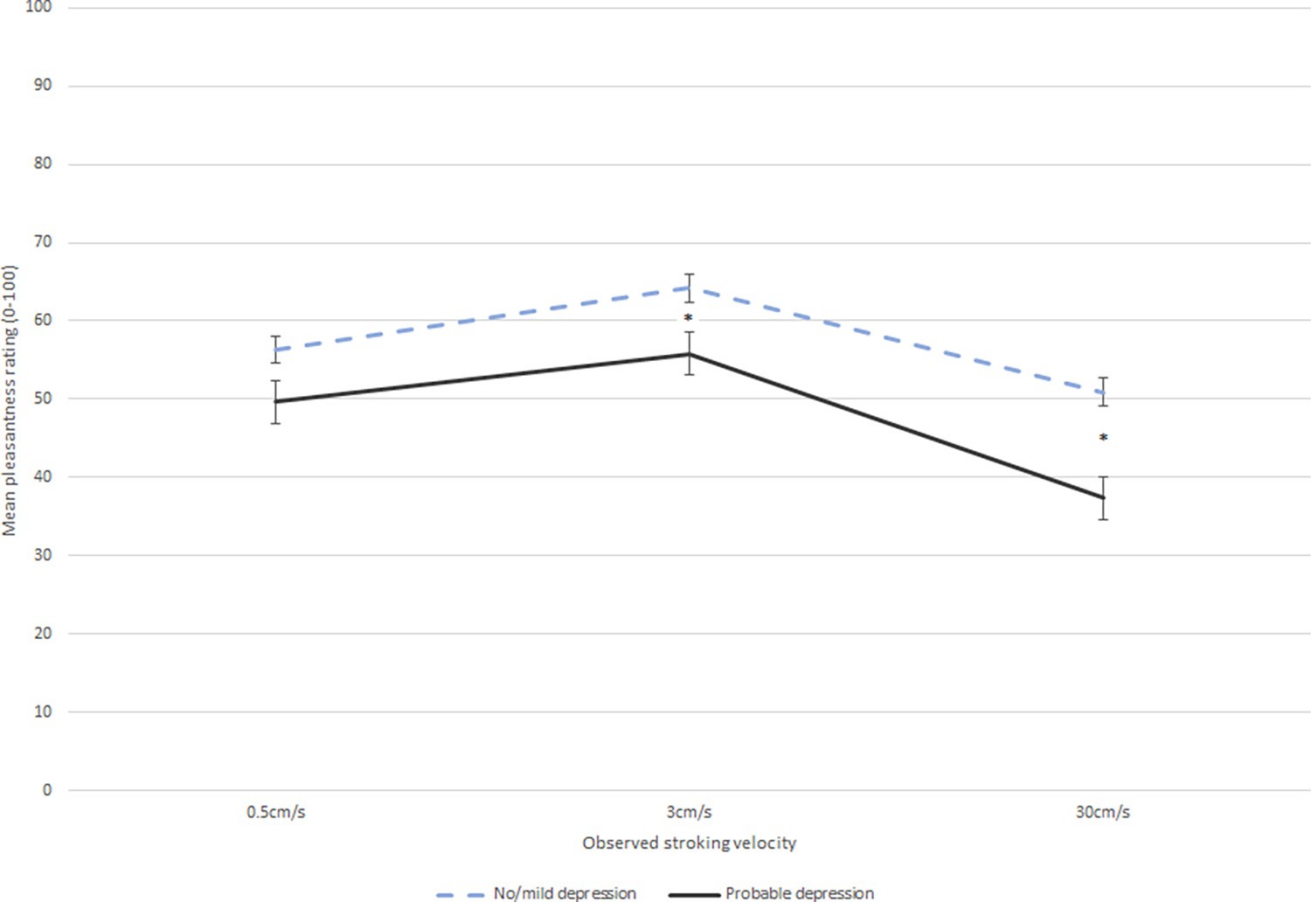
Interaction of stroking velocity and depression group on pleasantness ratings (controlling for general anhedonia and touch experiences and attitudes). Error bars show ±1 standard error of the mean. * = significant difference between groups at this velocity.

In an exploratory analysis (not pre-registered), we re-ran the above analysis and grouped participants into probable depression vs. no/mild depression. Results mirrored those above, with the probable depression group reporting significantly lower pleasantness ratings than the non/mild depressed group (*p* = .035; *M* = 57.13 and *SE* = 1.59 for no depression, and *M* = 47.61, *SE* = 2.51 for probable depression group). The effect of velocity was also the same as above. Interestingly, breaking down the significant depression-by-velocity interaction, the probable depression group rated the 30cm/s velocity as less pleasant than did the non/mildly depressed group (Bonferroni-adjusted contrast = -13.52, *p* < .001), and *also* rated the CT-optimal 3cm/s velocity as less pleasant than did the non/mildly depressed group (Bonferroni-adjusted contrast = -8.39, *p* = .049); there was no significant group difference for the 0.5cm/s velocity (Bonferroni-adjusted contrast = -6.64 *p* = .171); see Fig 2. Therefore, while depression severity influenced perceived pleasantness of observed touch, this was not specific to CT-optimal touch.

### Examining the association between touch longing and depression severity

A regression analysis with bootstrapping (1000 replications) was carried out to explore the association between depression symptoms and longing for touch. As the TEAQ_AUT subscale was not correlated with BDI-II score (see correlations), we did not include this subscale in the analysis. Bootstrapping was used as depression symptoms were not normally distributed. In step 1 of the regression analysis, the association between longing for touch and depression was not significant (*p* = .054, see Table 4), and longing for touch explained only 2% of the variance in depression symptoms (*R*^2^ = .02; Wald *Χ*^*2*^(1) = 3.72, *p* = .054). In step 2, we added general loss of pleasure and touch experience and attitudes into the model. The effect of longing for touch was still non-significant once these predictors were added, and we found that higher loss of pleasure and less current intimate touch were significantly associated with greater depression severity. At step 2, the model explained 34% of the variance (*R*^2^ = .34, *Wald χ*^*2*^(8) = 130.19, *p* < .001). Depression severity was therefore not significantly associated with a lower longing for touch, contrary to Hypothesis 2. However, it is interesting to note the association between current intimate touch and depression severity, which we return to in the discussion.

**Table 4:** Regression analysis reporting the association between longing for touch and depression severity.

	Outcome: BDI-II	*b*	*bootstrapped SE*	*p*	95% CIs	
Step 1	LITPQ	2.26	1.17	.054	-0.04	4.56
Step 2	LITPQ	1.46	0.93	.116	-0.36	3.29
	SHAPS	2.46	0.34	.000	1.79	3.12
	TEAQ_FFT	0.04	0.07	.632	-0.11	0.18
	TEAQ_CIT	-0.19	0.06	.004	-0.31	-0.06
	TEAQ_ChT	-0.14	0.09	.110	-0.32	0.03
	TEAQ_ASC	0.13	0.12	.265	-0.10	0.37
	TEAQ_AIT	0.03	0.08	.678	-0.12	0.19

Note. SHAPS = Snaith–Hamilton Pleasure Scale; TEAQ = Touch Experiences and Attitudes Questionnaire: FFT = Friends and Family Touch; CIT = Current Intimate Touch; ChT = Childhood Touch; ASC = Attitude to Self-Care; AIT = Attitude to Intimate Touch; LITPQ = Interpersonal Touch Picture Questionnaire.

## Discussion

We investigated the association between depression severity, perception of observed social touch, and attitudes towards social touch. We predicted that greater depression severity would be associated with reduced pleasantness ratings, especially for stroking in the ‘affective’ CT-optimal range (1-10cm/s) and CT-innervated location (forearm). Consistent with our hypothesis, we found that, across locations, higher levels of depression severity were associated with lower perceived pleasantness. However, contrary to our prediction regarding touch velocity, greater depression severity was linked to lower pleasantness ratings especially for the fastest non-CT-optimal velocity. Furthermore, while longing for touch was correlated with depression severity in bivariate correlations, this was no longer significant once we took general anhedonia and touch experiences and attitudes into account. Instead, lower current intimate touch was linked to greater depression severity.

While depression severity has been associated with a flattened U-shaped curve previously [31], we found that rather than a dampened response to viewing CT-optimal touch, greater depression severity was associated with reduced pleasantness for both CT-optimal and non-CT-optimal touch, and especially for the fastest non-CT-optimal velocity. As we controlled for general anhedonia, loss of pleasure in general cannot account for these effects, nor can individual differences in touch experiences and attitudes. It may be the case that individuals experiencing more severe depressive symptoms have a reduced desire to communicate [43–45], and therefore touch that is social in nature [21], regardless of velocity, is not rewarding or appealing [4, 5]. In addition, fast velocity touch has been found to communicate more negative emotions (such as fear) and intentions (such as warning; see [46]); perhaps this was accentuated in the context of greater depression symptoms, which may explain the lower pleasantness rating.

Previously, the location (palm vs. forearm) of affective touch has been shown to have a significant impact on perceived pleasantness, as there is a difference in CT innervation between these sites [11–14, 47]. The difference in ratings between glabrous and hairy skin is not a consistent finding, however [48]. In this study, no significant effect of location was found. This may be due to the vicarious nature of the stimuli, as participants were watching the stroking touch, rather than experiencing tactile input. Walker et al. [36], however, also explored vicarious social touch and did find a significant main effect of location, with touch on the back being rated as significantly more pleasant than any other location (upper arm, ventral forearm, dorsal fore- arm and palm). As we only included palm vs. forearm, it is possible that including the back as one location might have changed our results. In a further difference to our study, Walker and colleagues also did not control for the TEAQ or SHAPS in their analysis. Furthermore, effects of gentle stroking on pleasantness are also found in glabrous locations: perceived pleasantness when touch is delivered to glabrous skin may be due to a learned or secondary reinforcement mechanism underpinned by low-threshold mechanoreceptors [11, 49].

Regarding how much touch is desired, or longed for, we found indicators for an association between depression severity and touch longing, which was non-significant when we controlled for general loss of pleasure and touch experiences and attitudes. Rather than touch longing, general anhedonia and experiences or exposure to touch, especially current intimate touch, may better explain variance in depression severity. We found that levels of current intimate touch were significantly negatively associated with depression symptoms. As social withdrawal is a key feature of depression [1], withdrawing from social situations in which one might experience intimate touch might explain why current intimate touch is negatively linked to depression severity. However, if social withdrawal was the driving mechanism behind this association, then we would have expected to find an association between levels of friends and family touch and depression severity as well. While we did find this relationship in the bivariate correlations, the Friends and Family Touch subscale did not predict depression severity when accounting for the other TEAQ scales, suggesting that Current Intimate Touch better explains depression severity. The Current Intimate Touch subscale of the TEAQ includes items regarding stroking touch, which likely activates CT fibres more than the briefer, non-stroking touches included in FFT subscale [50]. The association specifically with the current intimate touch subscale may suggest that greater CT-targeted intimate touch is more protective against greater severity of depression. As our data is cross-sectional, we can only speculate as to causal mechanisms and the direction of causality (levels of current intimate touch as a precursor of consequence of depression), but we hope this study serves as an impetus for future research.

We should note that the study was conducted during the Covid-19 pandemic when restrictions were placed on physical contact with other people to reduce the spread of the virus. In the United Kingdom, physical contact was essentially limited to people sharing a household. In this sample, most participants lived with at least one other person, indicating some contact with others. Field et al. [51] conducted a survey exploring touch deprivation during the COVID-19 pandemic. Sixty percent of participants reported experiencing low to high levels of touch deprivation during the pandemic. Of these, 23% reported living alone, indicating that people who lived with others still experienced touch deprivation. During the pandemic, the public was also receiving messages about the negative effects of physical contact. A reduced exposure to touch combined with such public health messages may have resulted in an aversion to touch and, therefore, an overall reduced desire for touch. Without a comparison group of people tested outside the context of the pandemic, this is, however, difficult to verify.

This study had several limitations. Participants were predominantly Caucasian and female. This limits the generalisability of the findings in a multi-ethnic population, for example in the United Kingdom, where the study took place. The ethnicity of the individuals in the touch videos (all white) is also a limitation of this study. In addition, negative associations have been reported between depression severity and cognitive processes such as perspective taking and empathy [52–54]. It is possible that individuals with greater depression severity may have found it more difficult to consider how they themselves would find the touch if it was being delivered to them. Lastly, the cross-sectional nature of the study means we can only speculate about the direction of causality. Nevertheless, the association between depressive severity and reduced pleasantness as well as lower levels of current intimate touch warrants further investigation. It would be useful to replicate this study with delivering stroking touch to explore whether depression severity is associated with perceived pleasantness when the touch is directly experienced–and controlling for individual differences and loss of pleasure here as well. Furthermore, while we measured general anhedonia by self-report questionnaire, a task contrasting affective with neutral stimuli in another domain, such as presenting pictures, would enable us to understand whether any effects are touch-specific or more general in nature.

Interestingly, in intervention settings, touch has been shown to be effective in reducing depressive symptoms, for example in the form of aromatherapy massage [55] or Swedish massage [56] and with children and adults [57, 58]. Behavioural approaches to depression view withdrawal from usual activities as reducing access to positive reinforcement, leading to further withdrawal and increasing negative mood [59]. Therapeutic approaches such as behavioural activation encourage people to re-engage in activities that are potential sources of positive reinforcement. In non-depressed individuals, affective touch promotes social approach motivation [48]. Encouraging people to seek out touch, that is, increasing their exposure to touch, may in this way act as a positive reinforcer for social interaction and approach, and ultimately enhance the pleasantness of the experience.
